# Effect of the level of alveolar atrophy on implant placement accuracy in guided surgery for full-arch restorations supported by four implants: an in vitro study

**DOI:** 10.1186/s13005-023-00387-w

**Published:** 2023-08-30

**Authors:** Gökhan Göçmen, Ahmet Bayrakçıoğlu, Ferit Bayram

**Affiliations:** Department of Oral and Maxillofacial Surgery, School of Dentistry, Marmara University, Istanbul, Turkey

**Keywords:** Dental implant, Computer-aided designs, CAD-CAM, Edentulous jaw, Bone screws, Atrophy

## Abstract

**Background:**

A fixed screw-retained full-arch restoration supported by four implants is a popular treatment option for edentulous arches. Optimal alignment of implants is quite challenging in extremely atrophied edentulous cases, and a small amount of deviation is expected during guided surgery. This study aimed to compare implant accuracy among edentulous jaws with various levels of atrophy.

**Methods:**

Five separate copies of each Cawood and Howell model (III–V) were produced for the maxilla and mandible. A total of 120 implants (30 models). The implant accuracy was assessed based on angular deviations at the base (angle, 3D offset, distal, vestibular, and apical) and tip (3D offset, distal, vestibular, and apical).

**Results:**

The atrophy level of the jaws had a statistically significant effect on deviation; implants showed greater deviation from the planned location as the atrophy level increased.

**Conclusion:**

Given that implant deviation increased with the degree of atrophy, a greater safety margin from important anatomical structures is recommended when planning implant location for guided surgery in Cawood and Howell V cases.

## Introduction

The placement of dental implants in cases with limited alveolar bone volume is quite difficult. Cross-arch fixed dental prostheses, supported by four implants, are widely used for atrophied edentulous jaws. Efficient use of the existing bone is important; in this respect, 3D implant planning and guided surgery could be advantageous [1, 2].

Judicious intraoperative decision-making during implant site preparation is critical in cases of limited alveolar bone volume because of the proximity of important anatomical structures to the atrophied alveolar bone. There is a risk of inferior alveolar nerve damage and maxillary sinus membrane perforation. The use of longer implants that extend to the cortical borders of the alveolar bone is preferable to ensure bicortical anchorage and immediate stability to allow loading. In this scenario, guided surgery could be useful for placing implants [3, 4]; however, a small amount of deviation can occur even during guided surgery. The deviation between the planned and actual implant positions might cause problems in cases of bone atrophy, which can undermine adaptation and complicate the use of surgical guides. Various factors affecting the accuracy of guided surgery have been investigated, including the flap approach, type of support (tooth/mucosa), template position, fixation method, fabrication process, and implant insertion protocols [5, 6]. However, the effect of atrophy on guided surgery itself has not been investigated, despite its frequent occurrence.

The goal of this study was to compare the accuracy of guided surgery-placed axial and tilted implants based on the amount of bone loss in edentulous jaws. To our knowledge, this is the first study to investigate the effects of atrophy on implant accuracy.

## Materials and methods

The study was designed to evaluate the in-vitro accuracy of implant placement. Approval was obtained from our University Faculty of Dentistry Ethical Committee (approval no. 2018/157). Digital Imaging and Communication in Medicine (DICOM) data from a real clinical case were used to produce the base model. Materialise Mimics (Materialise Medical Software, Leuven, Belgium) was used to obtain standard tessellation language (STL) data. Input parameters were between 226 and 3,071 Hounsfield units. In the 3D models, the maxillary and mandibular regions were distinguished. The 3D printed models were made from Die and Model Resin ( Sprintray, Los Angeles, CA, USA). It has a 1700 mPa Flexural Modulus and 66,7 mPa Flexural Strength. The models are all designed to have a bone-like structure. The 3D printing process was performed with a layer height of 20 microns to ensure optimal model accuracy. In the Z direction, the outermost 4 mm is like cortical bone with 100% filling, and the inner part, the cancellous bone, is less with different filling patterns. All models are 3D printed in this way.

Meshmixer software (Autodesk, Mill Valley, CA, USA) was used to derive maxillary and mandibular models. The Cawood and Howell classification was used to differentiate among levels of atrophy [7]. Cawood and Howell III–V residual ridge types were designed for both the maxillary and mandibular models. Digital light processing technology (Moonray S 3D printer; Sprintray, Los Angeles, CA, USA) was used to print five copies of each design for the maxilla and mandible.

Initially, the gingival mask was constructed virtually on bone models with a 3 mm thickness for all bone surfaces. A pattern model was created to suit precisely these models containing the standard gingiva thickness. This model was designed to prevent sliding during slicon rubber weaving by fixing pin gaps at three distinct locations. On the same 3D printer, this gingival mask pattern mold was also produced. After affixing it to the bone models, RTV-2 silicone (Aydın Kompozit, Konya, Turkey) was poured in and allowed to cure for 24 h.

**Fig. 1:**
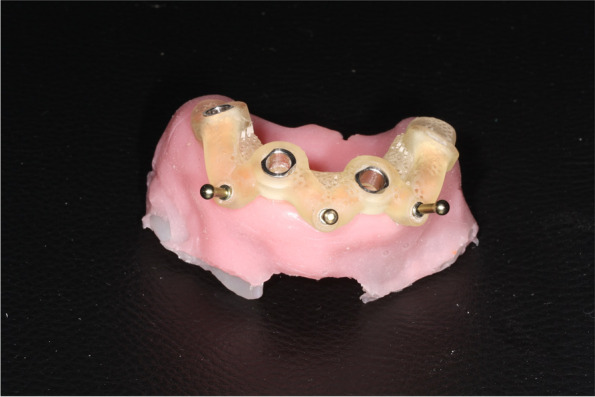
RTV-2 silicone rubber is used for the gum models, along with a surgical guide

The sample size was calculated with G*Power software (ver. 3.1.3; Heinrich-Heine Universität, Düsseldorf, Germany) based on an alpha value of 0.05 and statistical power of 90%. A sample size of 120 implants was required (20 implants per group). Five copies of each Cawood and Howell model (III–V) were reproduced for the maxilla and mandible. In total, 30 models were produced (Fig. 1).

**Fig. 2:**
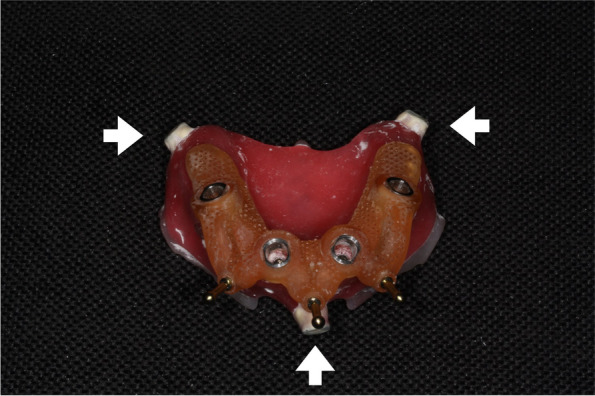
Radiopaque wax-covered screw heads are used to accurately superimpose STL data from the optical scan onto the DICOM data. STL, standard tessellation language; DICOM, Digital Imaging and Communication in Medicine

**Fig. 3:**
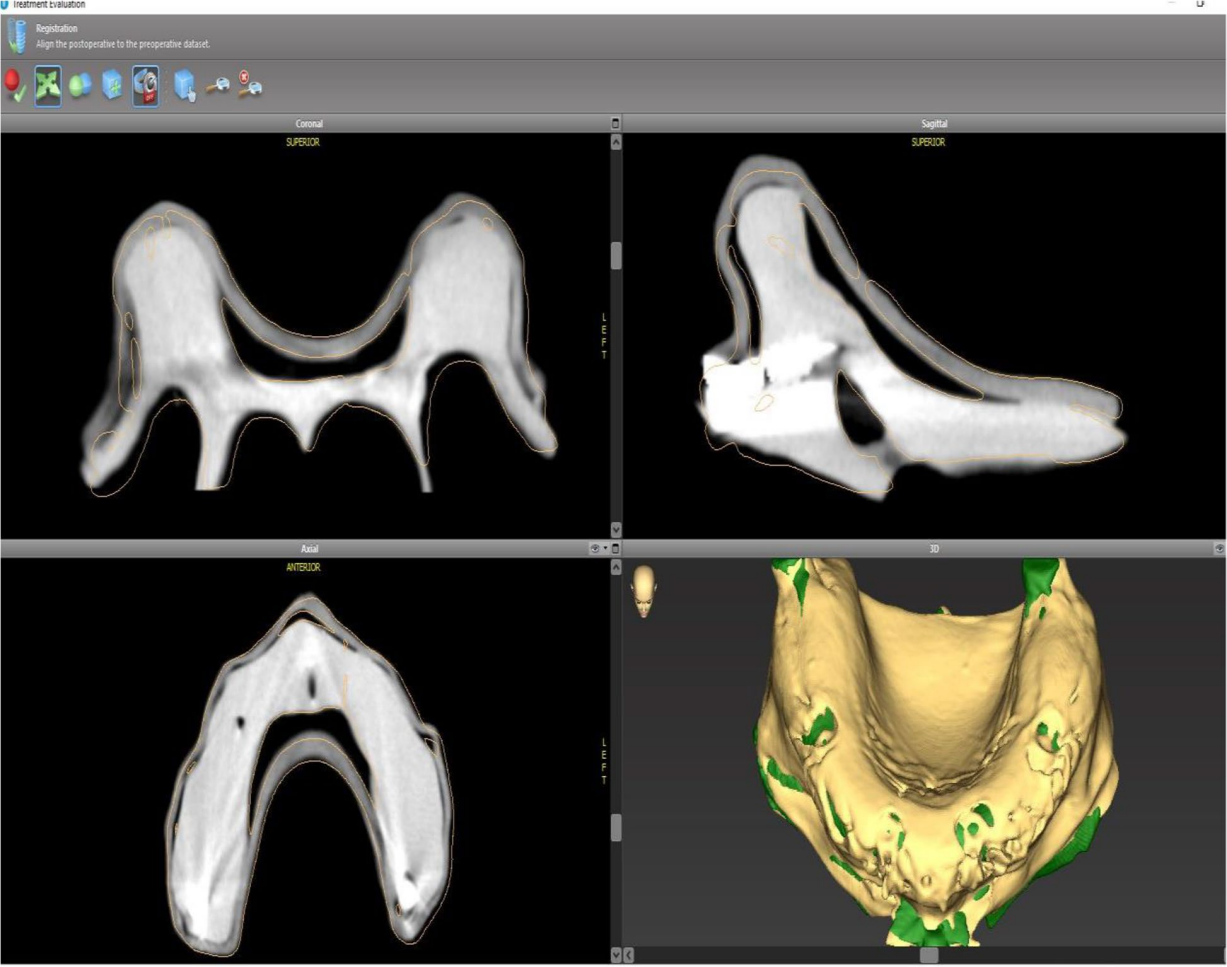
Superimposing STL data from the optical scan on the DICOM data in the coDiagnostiX software STL, standard tessellation language; DICOM, Digital Imaging and Communication in Medicine

Three 2.4-mm screws were placed to demarcate the anterior midline and posterior lateral lines on each side of the model. Computer-aided design/computer-aided manufacturing (CAD-CAM) wax (MarmoScan; Siladent, Goslar, Germany) was used to cover the screw heads and facilitate recognition during optical scanning (Fig. 2). We utilized a high-resolution cone-beam computed tomography machine (Planmeca Promax 3D Mid Dental Volumetric Tomography, Helsinki, Finland) to perform the CBCT scans. These scans were carried out using the subsequent parameters: 90 kV, 10 mA, and 36 s, with a Field of View (FOV) of 16 × 9 cm. White CAD-CAM spray (Dr. Mat, Istanbul, Turkey) was applied to the models to obtain higher-quality scans. The scanned gingival surface texture was transferred to the software of the NeWay optical 3D scanner (Open Technologies, Rezzato, Italy) (Fig. 3).

**Fig. 4:**
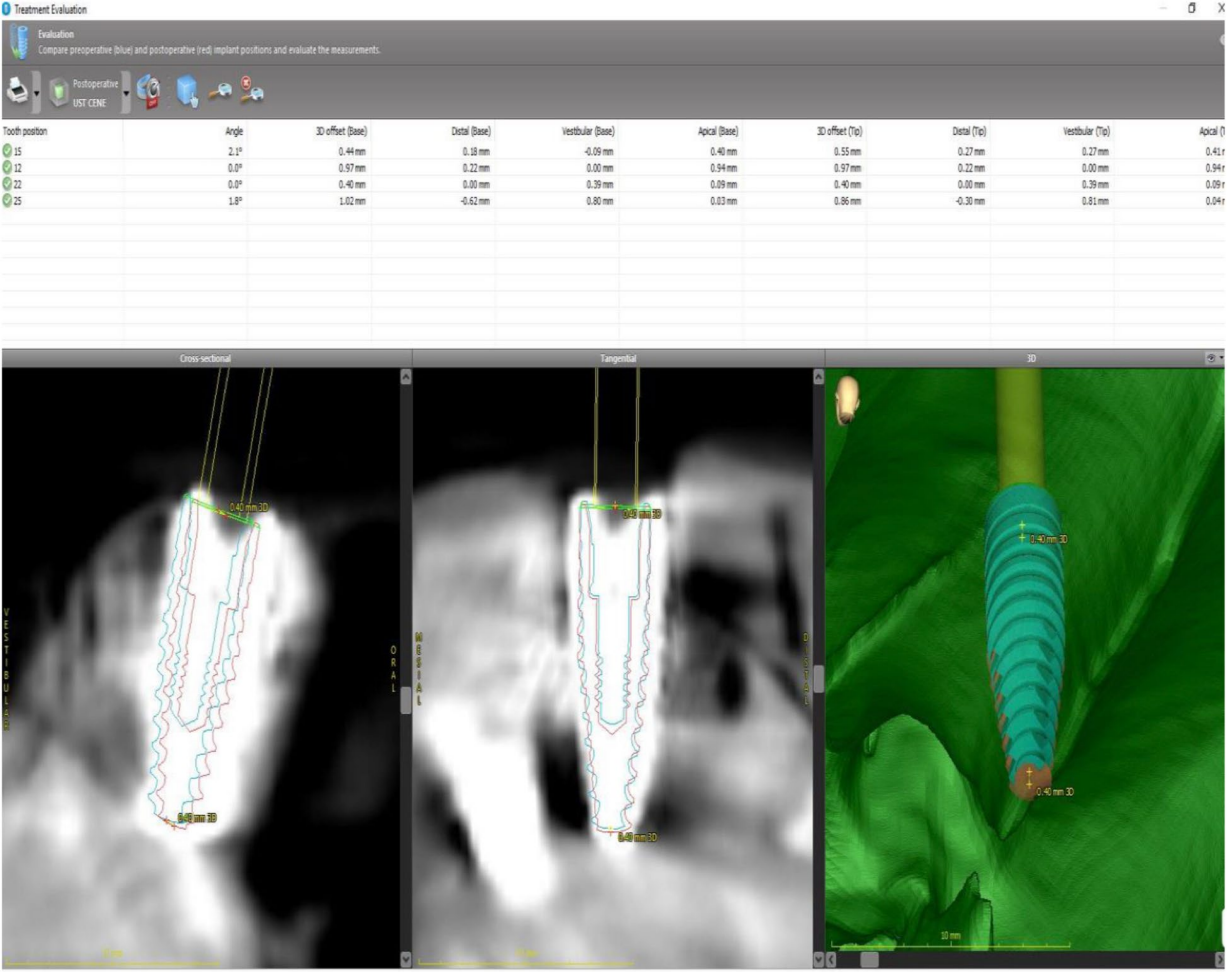
The comparison module in the coDiagnostiX software is used for comparing the planned and actual implant positions

coDiagnostiX software (Dental Wings Inc., Montreal, Canada) was used for implant planning and designing surgical guides. The radiopaque, wax-covered screw heads were used for accurate superposition of the STL and DICOM data. The gingival thickness of 3 mm on all surfaces and the cortical outer surface of 4 mm were measured and double-checked again on software, and models that did not have these features were not included in the study. After segmentation and marking of anatomical landmarks, virtual implants were positioned considering the available bone volume. Straumann (Basel, Switzerland) bone-level tapered implants (3.3 mm × 12 mm) were used in all regions. Four implants (two axial and two tilted) were planned for all models. In the mandibular models, the axial implants were located close to teeth #32 and #42. Two posteriorly angulated implants were positioned in front of the mental foramen at an approximately 30° angle. In the maxillary models, the axial implants were located close to teeth #12 and #22. Posterior angulated implants were placed in front of the anterior maxillary sinus wall at an approximately 30° angle. A constant anterior–posterior distance was maintained between the virtual implants. The surgical guide was designed using a sleeve of 5.0 mm in diameter and height. The guide design was sent to the laboratory and printed using the CARES P30 printer (Straumann). The entire process, from implant planning to surgical guide design, was overseen by a Straumann digital product consultant (Fig. 4).

During the interventions, the models were firmly fastened in a vise and stationed on a table to ensure stability and diminish variability throughout the procedure. A single operator performed all interventions, an accomplished oral surgeon with a track record exceeding ten years in the field of implant placement. The aim behind this was to mitigate any operator-dependent factors that could possibly affect the precision of implant placement. Surgical guides were fixed to the models using three pins passed through the sleeves (1.3 × 28 mm). The implants were placed in accordance with the recommended surgical protocol. Following implant placement, CBCT scans of the models were obtained. DICOM data were used to assess deviations from the planned locations. Marker screws were used for superimposition. The comparison module of the coDiagnostiX software was used for the assessment. Positional accuracy was evaluated by comparing the virtually planned and actual implant positions (Fig. 4). The implant placement accuracy was assessed based on angular deviations at the base [angle (A), 3D offset (B3D), distal (BD), vestibular (BV), and apical (BA)] and tip [3D offset (A3D), distal (AD), vestibular (AV), and apical (AA)].

Statistical analysis was performed using SPSS Statistics software (version 23.0; IBM Corp., Armonk, NY, USA). A two-way analysis of variance was conducted for the analysis of jaw shape and region. Multiple comparisons were made using Duncan's multiple-range test. The quantitative data are presented as the mean ± standard deviation. Statistical significance was set at *p* < 0.05.

## Results

There was a statistically significant effect of atrophy level on BV, BA, BD, AV, AA, and AD (*p* < 0.001). This significant result was due to higher average values for Cawood and Howell V than for Cawood and Howell III ridges (Table 1).

**Table 1:**
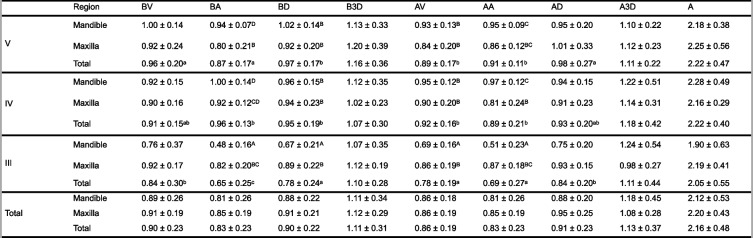
Comparison of the BV, BA, BD, B3D, AV, AA, AD, A3D, and A values according to atrophy level and region (maxilla or mandible). Data are presented as mean ± standard deviation

A–C: no difference between groups with the same le9er, A–D: no difference between inter-ac=ons with the same le9er *Abbreviations*: *A* Angle, *B3D* 3D offset, *BD* Distal, *BV* Vestibular, *BA* Apical, *A3D* 3D offset, *AD* Distal, *AV* vestibular, *AA* Apical

To gain a more nuanced understanding of these results, we evaluated the influence of both atrophy level and region (either maxilla or mandible) on these parameters. The analysis demonstrated significant effects of these factors on BA, BD, AA, and A (*p* < 0.001). Looking closely at the maxilla and mandible, in the Cawood and Howell V model, the implant at site 32 exhibited the highest mean BA and BD values of 1.04 and 1.13, respectively. In contrast, in the Cawood and Howell III model, the lowest mean BA value of 0.43 was observed for the implant at site 45, and the lowest mean BD value of 0.62 was seen for the implants at sites 32 and 42. Regarding AA, the highest mean value of 1.02 was noted for the implant at site 34 in the maxillary region of the Cawood and Howell V model. In comparison, the lowest mean AA of 0.4 was identified for the implant at site 42 in the mandibular region of the Cawood and Howell III model. For parameter A, the implant at site 44 in the maxillary region of the Cawood and Howell V model had the highest mean value of 2.74, while the implant at site 32 in the mandibular region of the Cawood and Howell III model displayed the lowest mean value of 1.52.

Implant location had no statistically significant effect on any parameter (Tables 2 and 3).

**Table 2:**
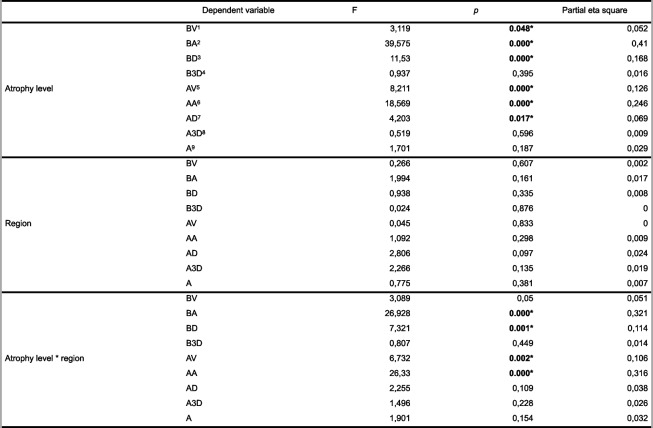
Comparison of the BV, BA, BD, B3D, AV, AA, AD, A3D, and A values according to atrophy level and region

A–C: no difference between groups with the same le9er, A–D: no difference between inter-ac = ons with the same le9er *Abbrevia tions*: *A* Angle, *B3D* 3D offset, *BD* Distal, *BV* Vesti bular, *BA* Apical, *A3D* 3D offset, *AD* Distal, *AV* Ves ti bular, *AA* Apical

**Table 3:**
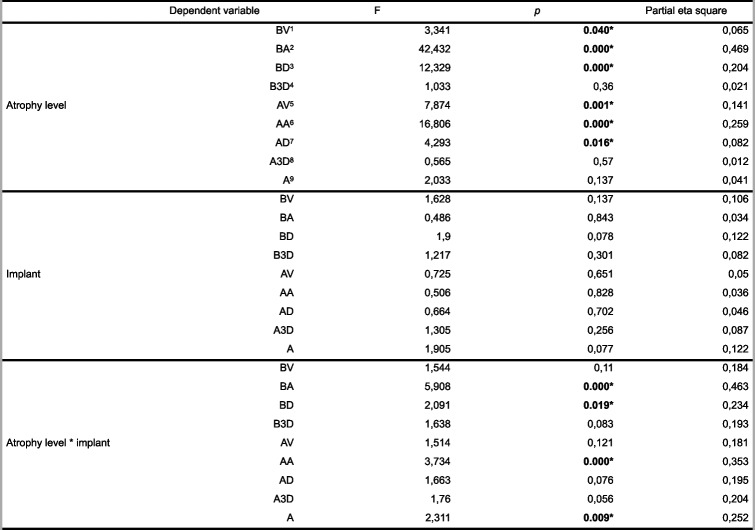
Comparison of the BV, VA, VD, V3D, AV, AA, AD, A3D, and A values according to atrophy level and implant

F: Analysis of variance statistic, 1R2 = 0.293, Adjusted R2 = 0.123; 2R2 = 0.640, Adjusted R2 = 0.554; 3R2 = 0.412, Adjusted R2 = 0.271; 4R2 = 0.259, Adjusted R2 = 0.081; 5R2 = 0.304, Adjusted R2 = 0.138; 6R2 = 0.482, Adjusted R2 = 0.358; 7R2 = 0.276, Adjusted R2 = 0.102; 8R2 = 0.267, Adjusted R2 = 0.091; 9R2 = 0.341, Adjusted R2 = 0.184 *Abbreviations*: *a* Angle, *B3D* 3D offset, *BD* Distal, *BV* Vestibular, *BA* Apical, *A3D* 3D offset, *AD* Distal, *AV* Vestibular, *AA* Apical. *P < 0.05

## Discussion

For clinical improvements in planning, design, and implementation, it is necessary to understand the limitations of each step of guided implant surgery to identify the reason for implant deviation from the planned position [8].

Careful positioning of implants near important anatomical structures and achieving immediate stability are important. In these respects, guided surgeries are facilitative, although deviations from the planned implant position still occur [9]. It is therefore important to analyze the factors that may increase this deviation. Deviations may be caused by errors during examination, planning, or surgery [5]. To minimize these errors, their origins must be identified [10]. Patient movement during the acquisition of 3D images may lead to image distortion, which can cause significant deviations at the apex and hex implant levels [11]. The thickness of the mucosa and the large maxillary surface area, which provide support for the surgical guide, can influence implant stability and placement accuracy [12]. It has been reported that tooth-supported guides provide better results than mucosa- and bone-supported guides [13]. The surgical technique, guide type, and implant position may also affect the accuracy of guided implant placement [14]. In our study, all other factors were controlled for, eliminated, or standardized, and only the effect of atrophy on deviation was examined. This is a limitation of our research. However, this is also our study's greatest advantage, as such restrictions and standardizations are not possible in clinical investigations.

Studies have shown that bone density can affect implant placement accuracy [15]. The degree of atrophy is related to bone density. As this was an in vitro study, it was not possible to assess the relationship between bone density and the extent of atrophy, which was a limitation of our study. However, all factors were controlled except for the atrophy level. All planning and surgeries were performed by the same individuals; all implants had the same diameter, length, and design; the same software was used throughout; and all models and guides were fabricated using the same 3D printer and resin. Therefore, the bone density can be considered consistent among all evaluations. The factors that we controlled for could not have been limited in clinical studies. The sophistication of the systems used in the evaluation of guidelines to measure sensitivity is also important. The guide system we used in our study is mucosa supported. In this guide system, hexagonal positioning to minimize the deviation in depth and special milling systems to prevent the deviation in depth are used. In clinical applications, these tools prevent the issue of remaining on the surface or drilling deeper, depending on the density of the bone. Since our study was an in vitro study, all these variables were standardized, and all models were produced with the same gingival and cortical thickness. These were also measured and checked a second time in the software, and models that did not meet the standards were excluded from the study [16, 17].

Various methods of evaluating implant deviations have been reported in the current literature. Direct comparison of the planned and actual implant locations was performed by superimposing the CBCT images or by transferring the implant positions to the software environment with the help of abutments [18]. Although comparison of pre- and post-procedure tomograms is useful, an additional tomogram is required specifically to measure implant deviation, which might be an ethical issue in clinical studies. However, this was not an issue in our in vitro study.

The effects of various factors on implant placement accuracy have been extensively investigated. Yeung et al. measured the placement accuracy with three implant systems [19]. They reported mean implant displacements of 0.02 ± 0.13 mm mesially, 0.07 ± 0.14 mm distally, 0.43 ± 0.57 mm labially, 1.26 ± 0.80 mm palatally, 1.20 ± 3.01 mm vertically in the mesiodistal dimension, 0.69 ± 2.03 mm vertically in the labio-palatal dimension, 1.69 ± 1.02° in terms of mesiodistal angulation, and 1.56 ± 0.92° in terms of labio-palatal angulation. The average displacements were similar between our study and a previous study [19]. An important issue for guided surgery in edentulous cases is the difficulty in accurately transferring mucosal thickness data into the software environment because of the lack of radiopaque structures (such as teeth) to guide superimposition [20]. In general, scans are used to estimate gingival thickness. In our study, we aimed to overcome the problem of transferring mucosal thickness data into the software environment by using screws as a guide. This approach can increase accuracy; we obtained satisfactory outcomes in terms of implant deviation, similar to those of implants placed using tooth-supported guides. The overall depth of the implant may deviate in mucosa-supported guides as a result of the gingiva's up-and-down flexion during drilling after the guide has been positioned. In order to prevent this clinical issue from influencing our study's results, we used models with equal gingival thickness and attached the guides to the models at three separate points. Even if there is some deviation as a result of this stretching, we do not believe it will have a substantial impact on the outcome, as it will occur at comparable rates across all models [21].

The maxillary surface area is wider than the mandible and provides greater support for surgical guides; this might enhance implant placement accuracy and stability [22]. However, the degree decreases with the progression of bone atrophy over time. In our study, implant deviations increased with increasing atrophy. However, no statistically significant difference was observed between the maxillary and mandibular implant deviations.

## Conclusions

We found that the level of alveolar atrophy was significantly associated with implant deviation. Some degree of deviation from the planned implant position is to be expected. The deviation observed in our study was similar to that observed in other studies. The outcomes were particularly similar to those of surgeries using tooth-supported guides. This could be due to our use of screws as a guide. Further studies on the clinical applications of these "superimposed screws" are merited.

In our study, no significant difference was observed in implant deviation between the upper and lower jaws. However, implant deviation increased with the degree of atrophy. Therefore, when planning the implant locations for guided surgery in Cawood and Howell V cases, a greater safety margin from important anatomical structures should be used. As this study was performed in vitro, further clinical studies are required on this subject.

## Data Availability

All data generated or analyzed during this study are included in this published article.
